# How to execute Context, Input, Process, and Product evaluation model in medical health education

**DOI:** 10.3352/jeehp.2019.16.40

**Published:** 2019-12-28

**Authors:** So young Lee, Jwa-Seop Shin, Seung-Hee Lee

**Affiliations:** Department of Medical Education, Seoul National University College of Medicine, Seoul, Korea; Catholic University, Korea

**Keywords:** Context, Input, Process, and Product model, Context, Input, Process, and Product evaluation model, Educational evaluation

## Abstract

Improvements to education are necessary in order to keep up with the education requirements of today. The Context, Input, Process, and Product (CIPP) evaluation model was created for the decision-making towards education improvement, so this model is appropriate in this regard. However, application of this model in the actual context of medical health education is considered difficult in the education environment. Thus, in this study, literature survey of previous studies was investigated to examine the execution procedure of how the CIPP model can be actually applied. For the execution procedure utilizing the CIPP model, the criteria and indicators were determined from analysis results and material was collected after setting the material collection method. Afterwards, the collected material was analyzed for each CIPP element, and finally, the relationship of each CIPP element was analyzed for the final improvement decision-making. In this study, these steps were followed and the methods employed in previous studies were organized. Particularly, the process of determining the criteria and indicators was important and required a significant effort. Literature survey was carried out to analyze the most widely used criteria through content analysis and obtained a total of 12 criteria. Additional emphasis is necessary in the importance of the criteria selection for the actual application of the CIPP model. Also, a diverse range of information can be obtained through qualitative as well as quantitative methods. Above all, since the CIPP evaluation model execution result becomes the basis for the execution of further improved evaluations, the first attempt of performing without hesitation is essential.

## Introduction

As times change and the education environment changes along with the students, education always possesses an unending possibility of change. Thus, experts are constantly contemplating on how medical health education can be improved. Effective improvements can be achieved when which aspects and how are determined. Thus, the suitable education evaluation method can facilitate the improvement of education.

Various education evaluation models exist depending on the meaning and perspective of the model and Worthen et al. [1] in 1997 categorized these models largely into objective-oriented, management-oriented, consumer-oriented, expertise-oriented, participant-oriented, and adversary- oriented evaluation models [1]. These approaches were summarized by Kim [2], Kim [3], and Sung [4].

The objective-oriented evaluation approach focuses on establishing goals in advance, and then on determining how far the goals have been achieved. However, the emphasis is only on evaluations of outcomes such as the effectiveness of education and student achievement, which can overlook the evaluation of teaching and learning process itself.

The management-oriented evaluation approach considers an evaluation to assist decision making by providing decision makers with the necessary information. The limitation is that it is rather complex to be fully implemented, but this approach allows the evaluator to assess all aspects of the program implementation. In addition, it helps to clarify the focus of the evaluation by helping the evaluator create important questions to be addressed at each stage.

The consumer-oriented evaluation approach regards everything used for education as an education product, and furthermore, conducts education as a service. Accordingly, attention is drawn to what consumers and consumers of education programs want and need. However, this approach can reduce the motivation of teachers and curriculum developers by considering only the consumer’s position.

The expertise-oriented evaluation approach is the oldest and most widely used model, and the method of evaluating education by expert judgment. The limitations of this approach can lead to irrational deliberations, since the weights of the criteria for the trivial and the important are not presented. In addition, manager prejudice can influence the formation of a review team.

The adversary-oriented evaluation approach is able to collect the opinions of the subjects broadly by dealing with all the opposing views in one evaluation, and help shed light on the advantages and disadvantages of the education program.

The participant-oriented evaluation approach attempts to take a holistic approach to humanistic issues in complex contexts and is characterized by value pluralism, and therefore can take a very different approach than other assessment approaches. But subjective or prejudiced interventions in the assessment cannot be ruled out. In addition, by excluding the evaluator’s role in the assessment, the evaluation method itself cannot assume the role of assessment.

Among these, the management-oriented approach provides the necessary information to the decision-maker to help the decision-making [4], and thus, this model type is appropriate in presenting the important information for education improvement.

Representative evaluation models are Alkin’s Center for the Study of Evaluation model and Stufflebeam’s Context, Input, Process, and Product (CIPP) model in the management-oriented approach. The CIPP model is a cyclic evaluation model [5] that can be modified at any time by detecting errors or deficiencies at each stage by providing information on decision-making about program planning, structuring, executing, and improving as well as evaluating activities. Therefore, the CIPP model is suitable for quality management of curriculum [6].

The CIPP evaluation model is most commonly used in the education field [6]. The main characteristic of this model is that the major objective of the evaluation is on improving rather than proving [7]. CIPP is an acronym for Context, Input, Process, and Product. Since evaluation is conducted using detailed criteria regarding these components, they are useful in carrying out systematic and structure evaluations [8].

In the world medical health professions education field, CIPP evaluation model is introduced and used for educational evaluation [9-16]. In addition, many educational sectors in Korea also use the CIPP evaluation model [5,6,9,17-26]. However, only a few studies can be found in educational evaluation in the field of medical health professions education in Korea [8,27]. As such, there are many advantages in evaluating education based on the CIPP model, but there is not much research and utilization in the field of medical education in Korea using the CIPP model. Although the complex characteristics of medical education go through a rather complicated process to implement the CIPP evaluation model [4,28], this study analyzes several previous studies and shows how they can be applied.

In this study, the experience of using the CIPP model to evaluate the “Medical humanities course” at Seoul National University College of Medicine and various previous studies were comprehensively investigated to determine how this model can be executed with what kind of procedure in the context of an actual medical education.

## Education evaluation execution procedure using the Context, Input, Process, and Product evaluation model

The following procedure is carried out when performing an evaluation using the CIPP model. First, the criteria and indicators are determined. Next, necessary materials and the method with which such materials will be collected for the evaluation are planned. Third, the collected materials are analyzed according to the criteria and indicators of each section of the CIPP model. Lastly, relationships between the CIPP sections are analyzed [8]. Recognizing how the CIPP model can be applied by following these evaluation steps can enhance the level of understanding.

## Development of Context, Input, Process, and Product evaluation criteria & indicators

When the ultimate goal of the evaluation is set, the very first step in the evaluation of education is determining the evaluation criteria and indicators. This step is also important in determining the direction of the evaluation. The evaluation criteria refer to the standard, principle, rule, or sign for the evaluation [29]. The criteria, which are the standard of the evaluation, facilitate communication between the evaluator and evaluation requestor regarding the evaluation subject or content based on the evaluation activity [17] as well as systematic judgment [18]. For these reasons, this step of determining the criteria and indicators has sufficient research merit in itself. The process of this step and its results have actually led to a significant research [17-21]. Likewise, the first step of evaluation requires substantial preparation and caliber as this step is significant enough to be regarded as an independent study subject.

The activity of setting the criteria and indicators is as follows. First, these steps are for when literature is used as the basis [8,9,20-22]: interview such as focus group interview [20,21], using the Delphi technique [19], and agreement between experts [8,10-12,19,22].

Understanding what each of the Context, Input, Process, and Product comprising the CIPP model means is important for a systematic evaluation. Context evaluation is the evaluation of the need, problem, asset, and opportunity within a situation [17]. Input evaluation assists in the decision-making of how facilities, human resources, and budget will be determined and constituted to achieve the goal of the education [27]. Process evaluation is the continuous examination of the program execution plan and process records [29]. The assessment of this step provides information regarding the schedule, method of progress, input activity type, and education method related to the education program to the education director so that this information contributes to the smooth progress fitting for the education goal [30]. Product evaluation measures and analyzes the results during and after the education [23] and examines the overall efficacy of the program [17]. This step has to inspect the intended effects, unintended effects, positive effects, as well as negative effects [24].

The CIPP model is employed as the evaluation method in various fields including science education, mathematics education, local education, education research and development, achievements through testing, education reliability of the government, school improvements, teacher training, human resource development, social welfare improvement, services of non-profit organizations, and technical development [25]. As the model can be utilized in various fields, the criteria can be diversely set according to the characteristics of the institution and program for evaluation, evaluation objective, evaluation context, and evaluation characteristics. Table 1 shows the criteria of literature and it can be observed that various criteria were used depending on the evaluation objective and field, which included institution evaluation, education evaluation, nursing education, and medical education.

However, the following common criteria can be obtained when content analysis was conducted for these criteria based on their frequency (Table 2): goals (6) and necessity or needs (5) had the highest frequency for context evaluation, material resources andfacilities (6), human resources (6), contents (5), and curriculum (3) for input evaluation, educational and service process (7), program evaluation (4), and educational courses and programs (3) for process evaluation, and finally, global satisfaction (5), students’ and service achievement (4), and program performance (4) for product evaluation. These results can become resourceful for future study and evaluations based on the CIPP model.

When evaluating a college on medical curriculum or education program, the education objective, achievement, focus, and operation guideline have to be considered [8]. Drawing up a blueprint by integrating these elements with each element of the CIPP model contributes to the systematic decision-making through the evaluation.

## Material collection method determination and material collection

The material collection method determination and material collection can be largely divided into the method for quantitative evaluation and the method for qualitative evaluation as shown in Table 1. Stufflebeam and Shinkfield [7] presented material collection methods possible for each CIPP element. For context, system analysis, survey study, literature survey, public hearings, interviews, diagnostic assessment, and the Delphi technique were presented; for input, available human and material resources, resolution strategy, design procedure, possibility and economic analysis, literature survey, pilot program survey, advocacy groups, and pilot attempt were presented; for process, procedural disorder identification and accidental disorder awareness, detailed information acquisition for scheduled decision-making, description of the actual process, continuous interaction with the program operation staff, and observation of their activities were presented; for product, operational definition and measurement of the performance standards and collection of judgments by interested parties were presented. These various techniques can be used as methods of material collection for actual evaluation, and among them, the methods presented in Table 1 were mainly used in published academic papers. Specifically, quantitative material collection is possible through questionnaire, literature survey, and grades. For qualitative material collection, the methods of short answer surveys, interviews, meeting minutes, curriculum, syllabus, and literature were used in previous studies.

As shown in Table 1, in many cases, students and professors were included as subjects of material collection. However, there were also many cases where various related people were included as subjects. In order to persuade various people, it is necessary to understand the relationships between various related people and their evaluation demands through multi-faceted evaluation methods [31].

## Collected material analysis and relationship identification

The CIPP evaluation model was developed with the purpose of providing systematic information for decision-making as a proactive evaluation from the very beginning. Thus, an evaluation is defined as a process for planning, obtaining, and providing useful information necessary for determining decision-making solutions [32]. In the CIPP evaluation model, 4 types of decisions are made to improve the evaluation subject and these decisions are planning, structuring, implementation, and recycling. Planning decision sets the objectives, structuring decision composes the procedural method necessary to achieve these objectives, implementation is a practical decision regarding the selected procedure, and recycling decision determines the continuation, termination, and modification of the program [24]. For these 4 decision types, the CIPP evaluation model proposed by Stufflebeam and Shinkfield [7] involved the goals, plans, actions, and outcomes of the core value of the program being examined and modified through the context evaluation, input evaluation, process evaluation, and product evaluation, respectively [17]. In detail, decision-making regarding objective determination, order of priority, and distribution guideline can be done through context evaluation. With regard to the selection of strategy for the program, the collected information can become a guideline and can be put into the design of detailed procedure through the input evaluation. Process evaluation contributes to execution guidelines and product evaluation contributes to guidelines for termination, continuation, modification, and initiation [7].

Meanwhile, the results of each element can not only be utilized for the improvement of the corresponding elements but also the relationship between them can be identified for improvement. In the 2019 study by Lee et al. [8], a need to actively reflect the demands and capacity of the students was observed in the context evaluation, and this impacted the input, process, and product aspects as well. In addition, the strategy in the input element was positive but there was a need to faithfully carry out the execution of the original plan in the process element. Another study in 2012 by Al-Khathami [11] showed that problems found in the process also affected the product. Likewise, since education is a continuing, single system, relationships between the elements can be identified to make improvements, and when the analysis for each CIPP model element is completed, important messages can be obtained when the relationships between the elements are determined.

## Conclusion

Up to now, various previous studies were investigated with a focus on the CIPP evaluation model and explored from a practical perspective on which procedures and methods were employed. These results showed that evaluations using the CIPP model, which can be considered rather difficult, can provide the basis for education improvement and no longer be considered a tough task.

With regard to the execution of this model, the setting of the criteria has to be emphasized once more. The model may not be able to address unplanned evaluation questions [13]. Thus, this setting of the criteria can act as a definitive evidence that determines whether an evaluation is successful.

Especially, the omission of evaluation of unset parts becomes more vulnerable for quantitative evaluations. In this regard, a method of material collection that can cover this criteria determination is collecting as much qualitative material as possible. These materials can contribute in obtaining a diverse range of opinions that quantitative materials cannot explain.

Rather than utilizing a single group such as the student group as the evaluation material collection source, having a balanced perspective of various interested parties regarding education can improve the reliability and validity of an evaluation, which can then be utilized as convincing base data.

In this study, even if there is any hesitation in using the CIPP evaluation model due to realistic resource limitations despite completely understanding the CIPP evaluation model and recognizing its importance, it is recommended that the attempt be carried through because the result of the evaluation conducted now can be used as the basis for determining the criteria and material collection method of a future CIPP evaluation to be carried out.

## Figures and Tables

**Table 1.** Literature regarding the criteria for each CIPP model element

Item	Author	Category	Context		Input	Process	Product
Proposal by Stuffelbeam, founder of CIPP Model	Stufflebeam and Shinkfield [7] (1985)	Criteria	Define institution situation; learner identification and demand inspection; search for demand satisfaction opportunity; problem diagnosis and determination of objective appropriacy		System capability, solution program strategy, design procedure of strategy execution, budget, schedule check	Flaw check or prediction of procedures in progress or the execution process; information provision for preplanned sequential decisions; report and judgment of events and activities regarding the execution	Collection of technology/judgment regarding achievements; linkage with information on the objective, situation, input, process; value and advantage analysis
		Material collection method	Utilization of systems analysis, survey study, literature survey, public hearings, interviews, diagnostic assessment, Delphi technique		Available human and material resources, resolution strategy, design procedure, possibility and economic analysis, literature survey, pilot program survey, advocacy groups, pilot attempt	procedural disorder identification and accidental disorder awareness, detailed information acquisition for scheduled decision-making, describe the actual process, continuous interaction with the program operation staff, and observation or their activities	Operational definition and measurement of the performance standards and collection of the judgments by the interested parties, qualitative/quantitative analysis
		Purpose	Necessary for decision-making regarding education objective and purpose when education begins (provide standard of change plans, performance judgment): use in the decision-making for education planning		Necessary for support resource, resolution strategy, and design procedure selection (provide the basis for change activity composition, execution process judgment basis): use in the decision-making for education structuralization	Necessary for program planning, procedure, and improvement; necessary for actual situation basis provision in performance analysis: use in the decision-making for education execution	Operational definition and measurement of the performance standard; collection of judgments by interested parties regarding the performance; qualitative/quantitative analysis: use in the decision-making for recycling
Institution	Jung and Moon [26] (2013)	Criteria	Service demand and situation, service objective domain		Budget, human resource management, facility and resource environment, service operation and content	Service activity, service satisfaction, service evaluation	Service application, service performance
School	Shin et al. [19] (2018)	Criteria	Demand analysis, objective setting		Execution plan (human resource, procedure, support system, etc.), performance detail	Program activity, program management and evaluation	Program performance (achievement, satisfaction, effectiveness)
Nursing	Kim and Son [27] (2017)	Criteria	Intention and necessity		contents of hospital introduction, senior nurses’ working experience	Composition and facilitation	Usefulness of the program, feeling involvement through activities, global satisfaction
		Material collection method	Questionnaire		Questionnaire	Questionnaire	Questionnaire
Medical health professions	Ashghali-Farahani et al. [14] (2018)	Criteria	Inappropriate infrastructure; unknown duties		Biomedical approach; incomprehensive curriculum; lack of professional NICU nursing mentors; inappropriate admission process of NICU students; lack of NICU skill labs	More emphasize on theoretical education; the overlap of credits with each other and the inconsistency among the mentor; ineffective assessment	Preferring routine work instead of professional job; tendency to leave the job; clitical incompetency of graduates; dissatisfaction of graduates
		Material collection method	Semi-structured interview; open question				
		Target of evaluation	NICU student, NICU graduate nurse, neonatologist, faculty member, nurse				
	Neyazi et al. [10] (2016)	Criteria	Goals, organization and management area	Interest and understanding of students towards field and labor market; faculty members; research and educational spaces and equipment		Student research activity; educational courses and programs, teaching and learning process; student progress evaluation; evaluated factors for graduates	Efficiency of research and educational programs, teaching and learning process to increase knowledge and job performance of graduates
		Material collection method	Researcher-made questionnaires inspired from the CIPP model and internal evaluation literatures				
		Target of evaluation	Students, graduates				
	Al-Khathami [11] (2012)	Criteria	Achievement of program goals; barriers to achieve goals, objectives, and needs	Alternative procedural design for: contents, academic sessions, hospital sessions, half day release sessions		Process involved in to learning activities; trainers; theoretical sessions; clinical sessions	Overall impression about the program; barriers to achieve goals, objectives, and needs; assessment tools; enjoyment; satisfaction
		Material collection method	Questionnaire (quantitative, qualitative)				
		Target of evaluation	Trainee				
	Yarmohammadian and Mohebbi [15] (2015)	Criteria	Human specialists and scientific services for needs of the local community	Head of department, faculty,		Activities of group manager, students, administrators	-
				students, curriculum, funding, training facilities		of library; scientific research and teaching–learning activities of faculty	
		Material collection method	Questionnaire				
		Target of evaluation	Directorates, faculty members, students, and library staff				
	Neyazi et al. [12] (2016)	Criteria	Goals, management, and organization area	Facility and spaces		Educational courses and programs, learning and teaching process; administration and financial; program evaluation	Graduates
		Material collection method	Questionnaire				
		Target of evaluation	Department head, faculty members, and library staff				
	Rooholamini et al. [9] (2017)	Criteria	Perceptions of learning; perceptions of teachers; academic self-perceptions; perceptions of the environment; social self-perceptions	Content of curriculum		The process of learning; process of teaching	Students’ performance; the process of teaching and learning
		Material collection method	Review of current evidence on integration; consultation with experts; Modified Dundee Ready Education Environment Measure (DREEM)	A researcher made questionnaire		1: researcher–made questionnaires for evaluating the quality of each integrated course; 2: researcher–made questionnaires for evaluating the quality of early clinical exposure	1: learner centered integrated basic science, portfolios; 2: brainstorming (students); 3: semi-structured interview (professors of basic sciences)
		Target of evaluation	Students, faculty and administrators	Faculties and curriculum committee		First and second year medical students	First and second year medical students; professors of basic sciences
	Lee et al. [8] (2019)	Criteria	Goals, necessity or needs	Available input resources (human and material resources); educational strategy		Implementation according to plan; evaluation of the program by students	Goal achievement; satisfaction of the curriculum
		Material collection method	Questionnaire, FGI, meeting minutes, syllabus, curriculum	Questionnaire, FGI, meeting minutes, time table		Questionnaire, FGI, meeting minutes, syllabus	Questionnaire, FGI, meeting minutes, grades
		Target of evaluation	Students, faculty				

CIPP, Context, Input, Process, and Product; NICU, newborn intensive care unit; FGI, focus group interview.

**Table 2.** Content analysis of previous studies in Table 1 according to the reference frequency for each Context, Input, Process, and Product model section

Item	Context	Input	Process	Product
Keywords extracted by content analysis (frequency)	Goals (6); necessity or needs (5); infrastructure (2); organization (2); management (2); intention (1); duties (1); barriers to achieve goals (1)	Material resources, facilities (6); human resource (6); contents (5); curriculum (3); funding (2); academic approach (1); admission process of students (1); interest and understanding of students (1); educational and strategy (1); implement plan (1)	Educational and service process (7); program evaluation (4); educational courses and programs (3); student progress evaluation (2); composition (1); facilitation (1); administration and financial (1); service satisfaction (1)	Global satisfaction (5); students’ and service achievement (4); program performance (4); efficiency of research and educational programs to increase knowledge and performance (1); barriers to achieve goals (1); the process of teaching and learning (1)

## Supplementary materials

Supplement 1. Audio recording of the abstract.
